# Honokiol suppresses the aberrant interactions between renal resident macrophages and tubular epithelial cells in lupus nephritis through the NLRP3/IL-33/ST2 axis

**DOI:** 10.1038/s41419-023-05680-9

**Published:** 2023-03-01

**Authors:** Qing Ma, Mengyang Xu, Xin Jing, Jiang Qiu, Shuo Huang, Honghao Yan, Lu Yin, Jiang Lou, Lisha Zhao, Yongsheng Fan, Ping Qiu

**Affiliations:** 1 The First School of Clinical Medicine, Zhejiang Chinese Medical University, Hangzhou, China; 2 School of Pharmacy, Guangdong Pharmaceutical University, Guangzhou, China; 3 Department of Clinical Laboratory, Tongde Hospital of Zhejiang Province, Hangzhou, China; 4 Department of Medicine, Hangzhou Normal University, Hangzhou, China; 5 School of Pharmaceutial Science, Zhejiang Chinese Medical University, Hang zhou, China; 6 School of Basic Medical Sciences, Zhejiang Chinese Medicine University, Hang zhou, China; 7 Department of Pathology, Affiliated Hangzhou First People’s Hospital, School of Medicine, Zhejiang University, Hangzhou, China; 8 Department of Pharmacy, Affiliated Hangzhou First People’s Hospital, Zhejiang University School of Medicine, Hangzhou, China; 9 Department of Medicine, Zhejiang Academy of Traditional Chinese Medicine, Hang zhou, China

**Keywords:** Autoimmunity, Drug delivery

## Abstract

Lupus nephritis (LN) is a type of immune-complex nephritis caused by systemic lupus erythematosus and is a major contributor to mortality and morbidity. Honokiol (HNK) has been found to have a therapeutic effect on LN, but its action mechanism remains unclear. In this study, we first demonstrated that HNK attenuates kidney injury in MRL/lpr mice. Results from RNA sequencing combined with ingenuity pathway analysis suggested that HNK plays an anti-LN role through inhibition of the NLRP3 inflammasome and IL33. GEO chip data, single-cell data, and clinical samples from LN patients demonstrated that the pyroptosis and IL-33/ST2 pathways are abnormally activated during the stage of LN. In vivo, similar to the results of the AAV-mediated NLRP3 shRNA MRL/lpr model, HNK downregulated serum and renal IL-33 levels, and suppressed NLRP3 inflammasome and the IL-33/ST2 axis in the kidney. In vitro, co-culturing NLRP3-overexpressing or IL-33 knocked-down rat renal macrophages with NRK-52E cells confirmed that NLRP3 activation in resident macrophages directly upregulates IL-33, which in turn mediates the IL-33/ST2/NF-κB pathway to promote the inflammatory response of renal tubular epithelial cells. Furthermore, a molecular docking model and surface plasmon resonance analysis were utilized to demonstrate a direct interaction between HNK and NLRP3. In conclusion, this study provides a novel anti-LN treatment strategy in which HNK plays a preventive and therapeutic role against LN by suppressing the abnormal crosstalk between renal resident macrophages and renal tubular epithelial cells by inhibiting the activation of the NLRP3/IL-33/ST2 axis.

## Introduction

Systemic lupus erythematosus (SLE) is a typical autoimmune disease and also a heterogeneous disease [1]. The adjusted worldwide prevalence of SLE is 50–100 per 100,000 adults and is tenfold more prevalent in childbearing women than in men [2, 3]. According to clinical statistics, lupus nephritis (LN) is the most common complication of SLE and poses a considerable risk of progression into end-stage renal disease, which is a major cause of death [4]. Despite the increasing understanding of the pathogenesis of LN and therapeutic improvements, LN remains a major risk factor for incidence and mortality in SLE patients [5]. Therefore, efficacious complementary or alternative therapies with few side effects are urgently needed.

Traditional Chinese medicine (TCM) involves thousands of years of clinical experience in treating diseases based on a holistic concept and has received widespread attention because of its high efficacy and few side effects. Previous studies have shown that TCM-based treatments can improve the survival rate of SLE patients and reduce the prevalence of renal and various chronic diseases [6]. *Magnolia officinalis*, is a magnolia species that has been widely used as a herbal medicine in Japan and especially in China, where it has been clinically used for thousands of years [7]. Honokiol (HNK) is a pleiotropic lignan isolated from the bark of *M. officinalis*, and has various pharmacological actions, such as anti-inflammatory, antioxidant, and anti-tumorigenic activities, without noticeable side effects [8]. Previous studies have demonstrated that HNK has a therapeutic effect on an accelerated, severe form of LN in a mouse model, called NZB/WF1 mice [9]. In addition, HNK has also been shown to protect renal function, and have anti-inflammatory, and anti-injury effects in various cases of nephropathy, such as acute kidney injury [10], renal ischemia-and-reperfusion injury [11], and chronic renal inflammation and fibrosis [12]. However, the mechanism whereby HNK ameliorates LN remains elusive.

Currently, the high-throughput platform represented by bioinformatics can systematically analyze changes in in vivo gene expression, and this strategy has become an important means to study the mechanisms underlying the bioactivities of TCM-based therapeutics [13–15]. Ingenuity pathway analysis (IPA) relies on a strong background library to create customized pathways or interaction networks focused on molecular targets and thereby enables exploratory studies on genes, proteins, and biological functions. The powerful data-analysis capabilities of IPA help to understand the complex data in biological studies and facilitate the identification of potential biomarkers for disease diagnosis and treatment [16]. Accordingly, this study aimed to reveal the anti-LN mechanism of HNK by conducting integrated research at the cellular and molecular levels, with the ultimate aim of finding new targets and strategies for the development of anti-LN therapeutics.

## Results

### HNK reduced the renal injury and pathological changes in MRL/lpr mice

**Fig. 1:**
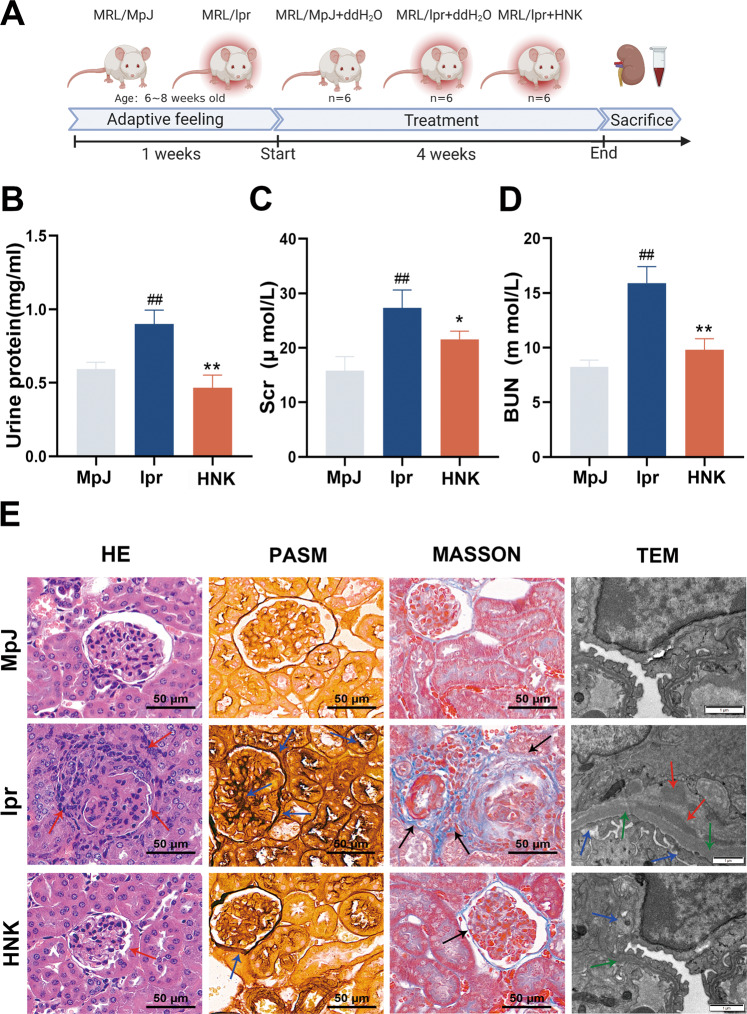
Honokiol (HNK) reduced renal injury and pathological changes in MRL/lpr mice. **A** The procedure of the animal experiments. **B** Urinary protein levels. **C** Serum creatinine levels. **D** Blood urea nitrogen levels. **E** Renal histopathological analyses revealed peri-glomerular inflammatory-cell infiltration (hematoxylin-and-eosin staining), glomerular mesangial hyperplasia (periodic acid silver methenamine staining), and mesangial fibrosis (Masson staining); 400×; scale, 50 μm. The red and blue arrows indicate inflammatory cells and intraglomerular mesangial proliferation, respectively. The black arrows indicate collagen fiber deposition. Shown are representative transmission electron microscopy images. Original magnification 15,000×; scale, 1 μm (*n* = 6, */#*p* < 0.05, **/##*p* < 0.01; # versus the MPJ group; * versus the lpr group).

The overall experimental scheme is presented in Fig. 1A. The results showed significant reductions in urinary protein, Scr, and BUN levels in the HNK group, as compared with the levels in the lpr group (Fig. 1B–D). HE staining showed that compared with the MpJ group, the lpr group had glomerular swelling, tubular atrophy, and high lymphocyte infiltration around the blood vessels. However, compared with the lpr group, the HNK group had a relatively normal glomerular structure with a significant reduction in the number of inflammatory-cell infiltrates in the renal interstitium. Similarly, results from PASM and Masson’s staining suggested that HNK reduced intraglomerular mesangial proliferation and collagen deposition. In addition, results from transmission electron microscopy (TEM) suggested that after the HNK treatment, the uneven thickening of the glomerular basement membrane (GBM), the fusion of podocyte foot process, and the electron-dense deposition in the GBM were improved. (Fig.1E).

### NLRP3 and IL33 were predicted to be the key targets of HNK in alleviating LN

**Fig. 2:**
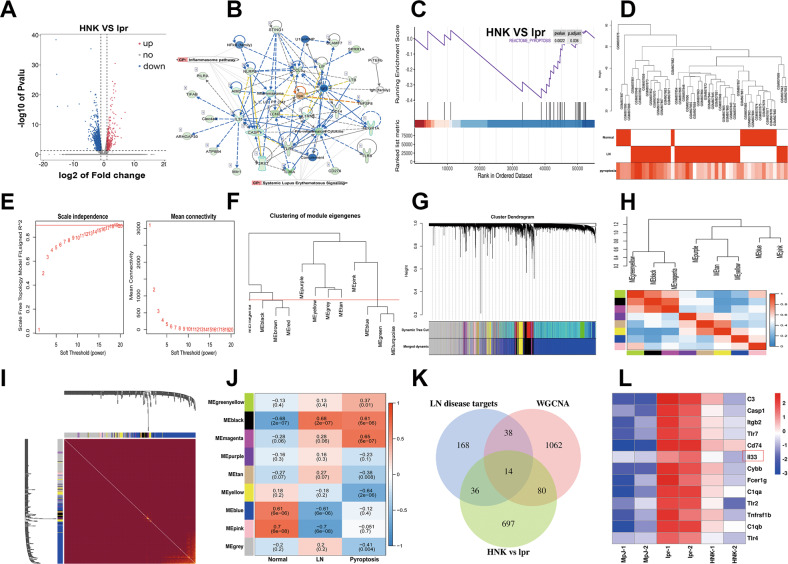
Changes in the overall gene-expression profiles after treatment with Honokiol (HNK). **A** Volcano plot illustrating the differentially expressed genes between HNK and lpr groups. **B** Gene interaction network after HNK intervention, based on ingenuity pathway analysis. **C** Gene set enrichment analysis of the HNK-induced differentially expressed genes related to the pyroptosis pathway. **D** Sample dendrogram and trait heatmap. **E** Scale-free topology model fit index and mean connectivity under various soft thresholds power, The cut-off soft threshold β was set as 0.9 and β = 15 was selected. **F** Clustering of module eigengenes with a shear height of 0.25. **G** Cluster dendrogram, showing the original modules and the combined modules. **H** Eigengene dendrogram and Eigengene adjacency heatmap of various modules. **I** Network heatmap plot of the co-expression modules. **J** Heatmap of the module-trait relationships. **K**, **L** A potential disease target of HNK ameliorates lupus nephritis (LN).

To gain insight into the action mechanism of HNK, renal gene expression profiles were evaluated using RNA sequencing (RNA-seq). The renal DEGs (*p* value <0.05 and |log2 (fold change) |≥2) between the HNK mice and lpr mice were identified (Fig. 2A) and results from IPA suggested that the NLRP3 inflammasome pathway was significantly enriched upon HNK treatment, and *IL33* was the core gene of the gene interaction network (Fig. 2B). Results from gene set enrichment analysis (GSEA) showed that the pyroptosis pathway was enriched among the genes downregulated upon HNK treatment (Fig. 2C). To identify the DEGs associated with the pyroptosis pathway, we selected 15 normal renal samples and 32 LN samples from the GSE32592 LN-patient dataset of the Gene Expression Omnibus (GEO) database and clustered them according to the different phenotypes of normal, diseased, and pyroptosis (Fig. 2D). Then, a weighted gene co-expression network was constructed (Fig. 2E–G), and the reliability of the module description was proven via a module correlation analysis (Fig. 2H, I). Subsequently, the module was correlated with the phenotypic data (Fig. 2J), and the black module, which is highly correlated with the three phenotypes, was identified. Finally, we intersected the genes in the black module gene with the DEGs and four LN-target databases (Fig. 2K), whereby we obtained 14 key genes highly related to HNK intervention, LN, and the pyroptosis pathways (Fig. 2L).

**Fig. 3:**
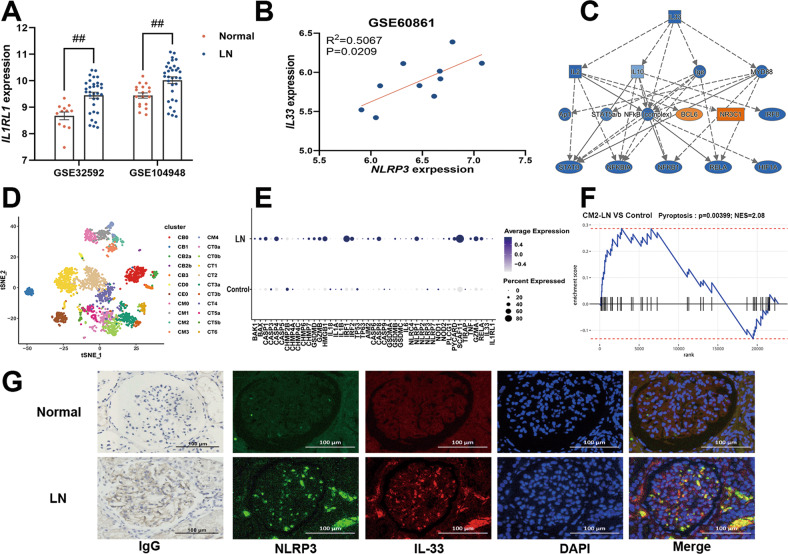
Bioinformatics analysis showed that the IL-33/ST2 and NLRP3 pathways were activated in LN. **A** *IL1RL1* was significantly upregulated in patients with lupus nephritis (LN). **B** A significant positive correlation between *NLRP3* and *IL33* was observed in patients with LN. **C** The factors downstream of IL-33 in the pathway activated by Honokiol, as predicted using ingenuity pathway analysis. **D** Clustering diagram showing the spatial dimensionality reduction of the single-cell data. **E** Bubble chart showing the pyroptosis-related genes, NF-κB, IL-33, and IL1RL1. **F** Gene set enrichment analysis of the single-cell pyroptosis pathway. **G** Immunohistochemical assessment of the IgG levels in the healthy and LN kidney samples and immunofluorescence staining of NLRP3 and IL-33. 200×; scale, 100 μm.

### Bioinformatics analysis showed that the IL-33/ST2 pathway and NLRP3 inflammasome were activated in LN patients

We downloaded the microarray gene expression data involving glomerulus and renal tubules from the NCBI GEO database and observed that *IL1RL1* was significantly upregulated in LN patients (Fig. 3A). In addition, there was a significant positive correlation between *NLRP3* and *IL33* (Fig. 3B), and our IPA results suggested that NF-κB was downstream of IL-33 (Fig. 3C). Next, we used single-cell sequencing-data analysis to explore the roles of the NLRP3 and IL-33/ST2 pathways in the pathogenesis of LN. To this end, we downloaded the single-cell sequencing data pertaining to LN and applied the UMAP algorithm to perform spatial dimensional-reduction clustering (Fig. 3D). Notably, the pyroptosis-related genes, *NF-κB*, *IL-33*, and *IL1RL1* were significantly upregulated in the LN patients, compared with the levels in the control group (Fig. 3E), and GSEA results further demonstrated that the pyroptotic pathway was significantly active in CM2 cells (tissue-resident macrophages) (Fig. 3F). In addition, immunohistochemical analysis for IgG expression and immunofluorescence co-staining for IL-33 and NLRP3 expression (Fig. 3G) revealed significant IgG deposition and upregulation of NLRP3 and IL-33 in the kidneys of the LN patients. Taken together, these results suggest the activation of the NLRP3 and IL-33/ST2 pathways in the renal resident macrophages of LN patients.

### HNK suppressed the renal activation of the IL-33/ST2 pathway and NLRP3 inflammasome in MRL/lpr mice

**Fig. 4:**
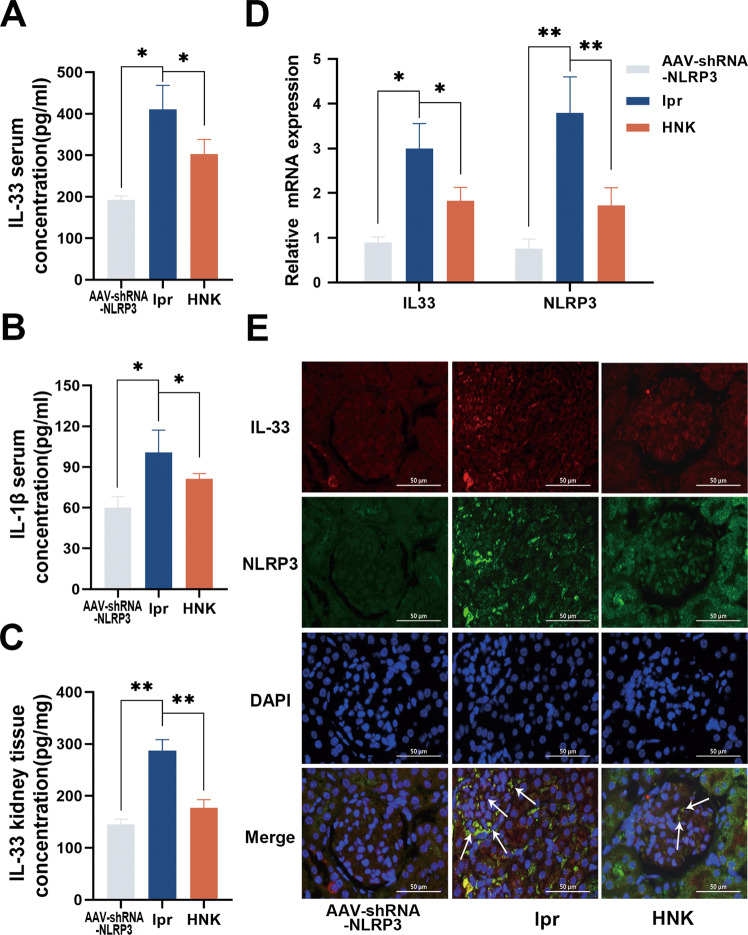
Honokiol (HNK) suppressed the renal activation of the NLRP3 inflammasome and IL-33/ST2 pathway in MRL/lpr mice. **A** Serum IL-33 levels. **B** Serum IL-1β levels. **C** Renal IL-33 levels. **D** HNK decreased the mRNA levels of *IL33* and *NLRP3* in MPJ/lpr mice. **E** Immunofluorescence co-staining for ST2 and NLRP3. 400×; scale, 50 µm (*n* = 6. *p < 0.05, **p < 0.01).

**Fig. 5:**
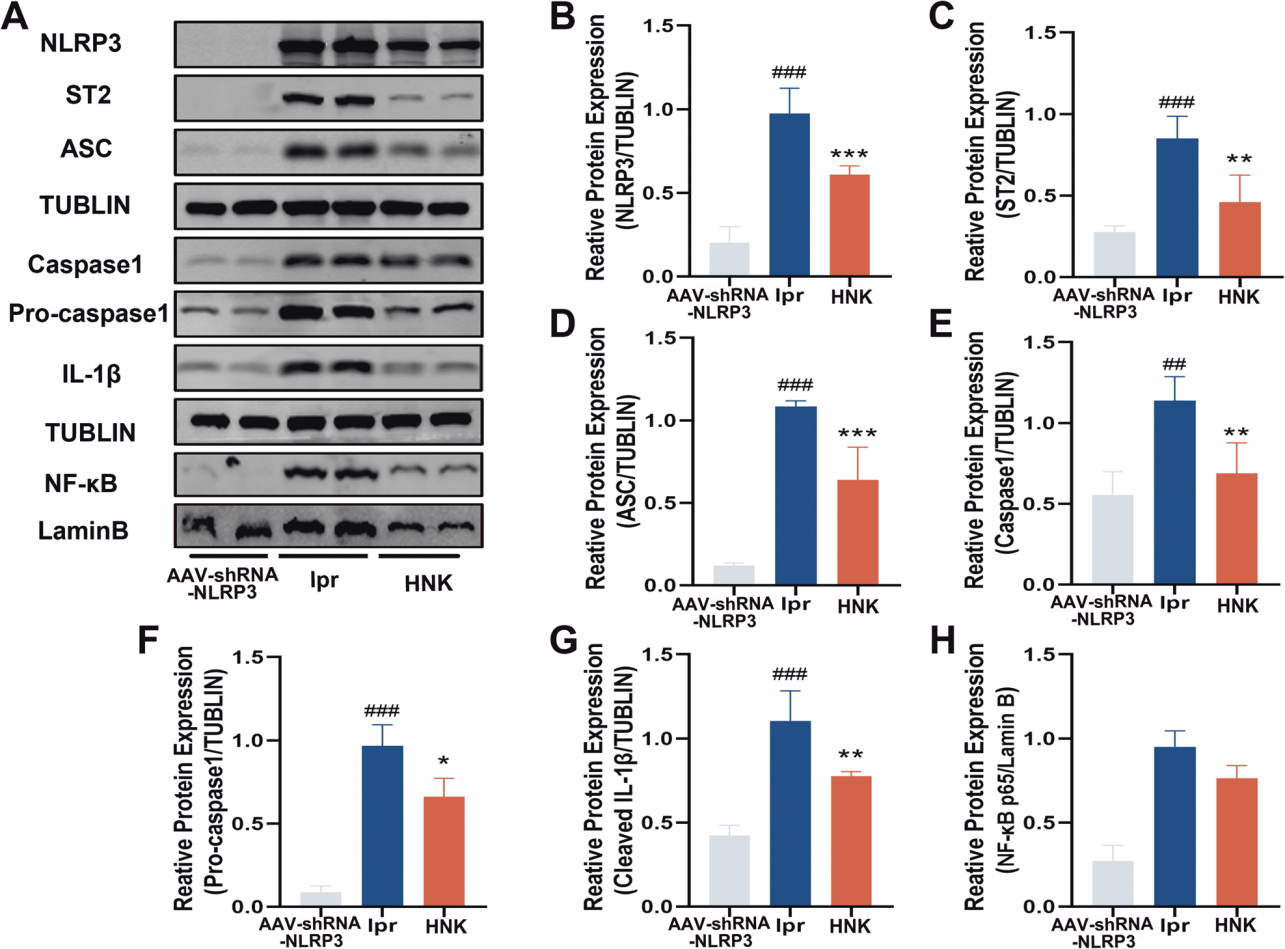
Effects of Honokiol (HNK) on the renal levels of the NLRP3 inflammasome and the ST2 and NF-κB proteins in MpJ/lpr mice. **A** Renal levels of the NLRP3 inflammasome, ST2, and NF-κB. **B–H** Gray-scale analysis (*n* = 3, */#*p* < 0.05, **/##*p* < 0.01; # the lrp group versus the AAV-shRNA-NLRP3 group; * the HNK group versus the lpr group).

To verify the bioinformatics results, we carried out in vivo experiments. ELISA results showed that the serum IL-33 and IL-1β and renal IL-33 levels in the lpr group were significantly decreased upon HNK treatment (Fig. 4A–C). In addition, HNK significantly decreased *NLRP3* and *ST2* mRNA levels compared with the levels in the lpr group (Fig. 4D). Immunofluorescence co-staining results also showed that the expression levels of IL-33 and NLRP3 in the HNK group were lower than those in the lpr group (Fig. 4E). Additionally, western blot results showed that NLRP3, cleaved caspase-1, caspase-1, cleaved IL-1β, ASC, ST2, and NF-κB were significantly downregulated in the HNK group, compared with the levels in the lpr group (Fig. 5).

### HNK suppressed the NLRP3-Induced IL33 upregulation in renal resident macrophages

**Fig. 6:**
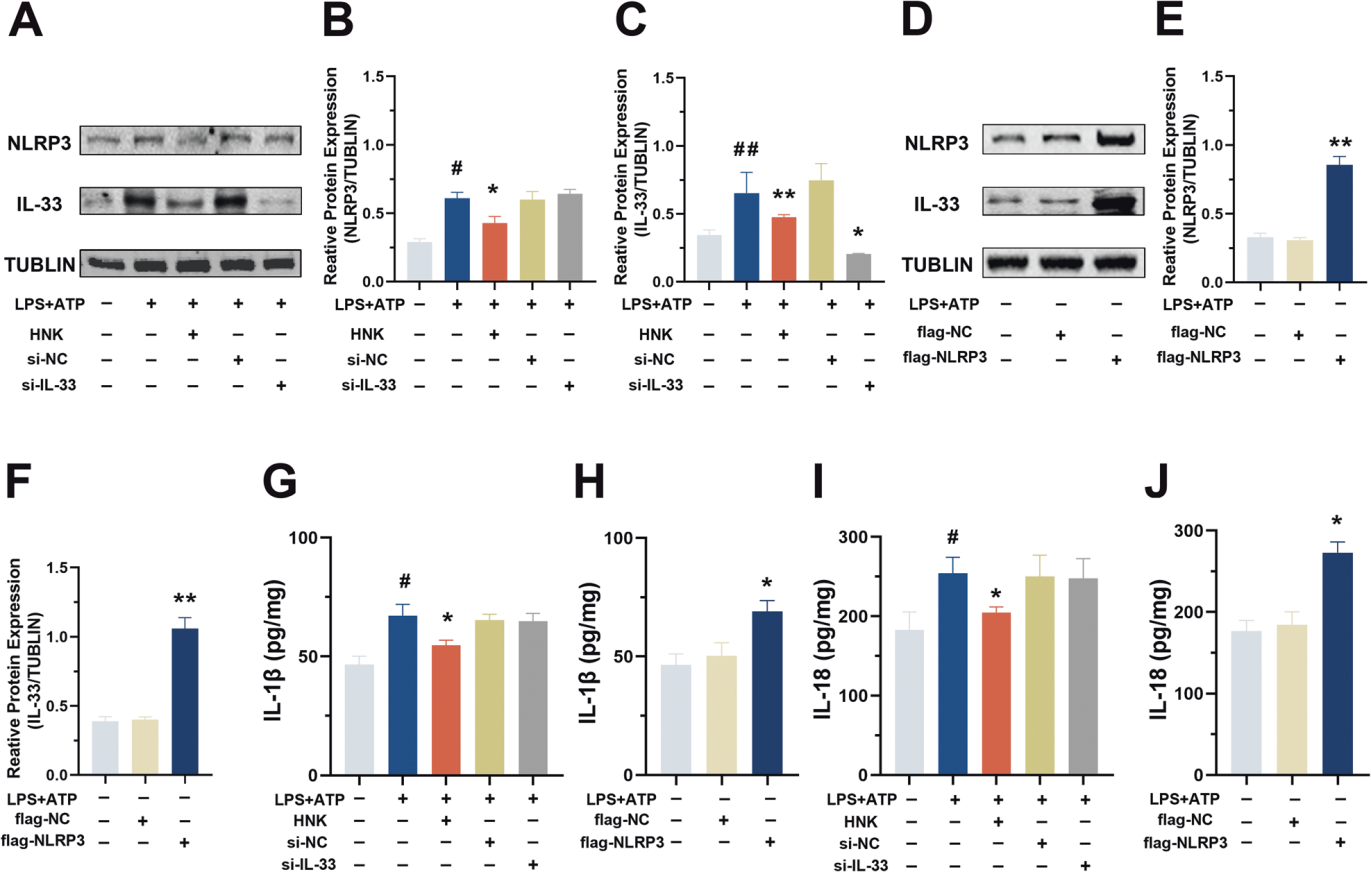
NLRP3 regulates IL-33 expression in renal resident macrophages. **A–F** Protein levels and gray-scale analysis of NLRP3 and IL-33 (*/#*p* < 0.05,**/##*p* < 0.01; #versus MPJ group; * versus lpr group). **G–J** IL-1β and IL-18 levels in the cell-culture supernatant (#*p* < 0.05, versus the blank control; *p < 0.05, versus the LPS + ATP group).

We found that the NLRP3 and IL-33 mRNA and protein levels in MPL/lpr mice were increased, compared with the levels in the adeno-associated virus (AAV)-shRNA-NLRP3 group. Single-cell sequencing results further suggested that the NLRP3/IL-33 axis was perturbed in the renal resident macrophages of LN patients. Furthermore, the GEO data showed that NLRP3 level had a significant positive correlation with IL-33 level in LN patients. To explore whether there is a regulatory relationship between the two, we used renal resident macrophages from rats. Previously, it has been shown that co-treatment of cells with the pro-inflammatory stimulator LPS and the oxidative stress product ATP can effectively activate the NLRP3 inflammasome [17]. We found that when the cells were treated with LPS and ATP, the protein levels of NLRP3 and IL-33 in the cells (Fig. 6A–C) and those of IL-1β and IL-18 in the cell-culture supernatants (Fig. 6G, I) were significantly increased, but they were all significantly decreased upon HNK treatment.

Next, we transfected rat renal resident macrophages with an NLRP3-expression plasmid or IL-33 siRNA and observed that the IL-33 knockdown had no significant effect on the cellular level of the NLRP3 protein (Fig. 6A–C), whereas overexpression of NLRP3 significantly increased the cellular level of the IL-33 protein (Fig. 6D–F) and the levels of IL-18 and IL-1β in the culture supernatant (Fig. 6G–J). Overall, our results suggest that NLRP3 directly regulates the expression of IL-33 in renal resident macrophages.

### HNK-mediated remodeling of the NLRP3/IL33/ST2 axis modulates the crosstalk between renal resident macrophages and renal tubular epithelial cells in LN

**Fig. 7:**
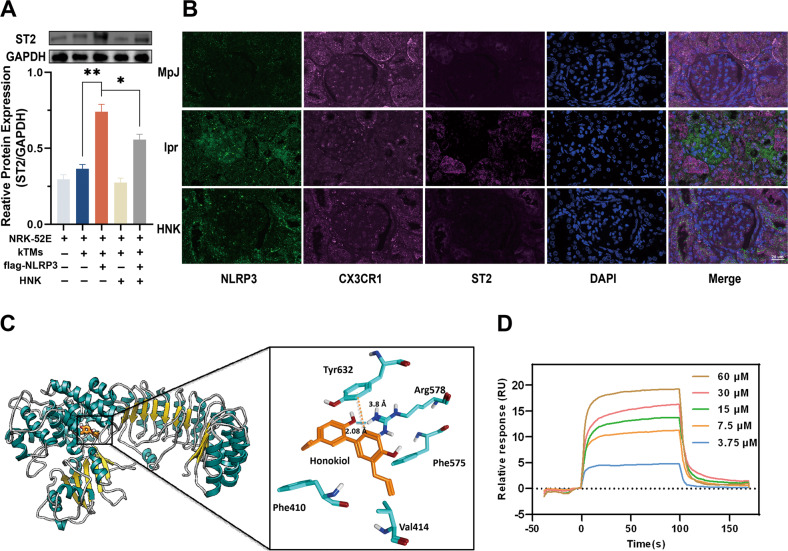
Remodeling of the NLRP3/IL33/ST2 axis by Honokiol (HNK) modulates the crosstalk between renal resident macrophages and renal tubular epithelial cells in lupus nephritis. **A** Co-culture of kTMs and NRK-52E cells, and protein levels in NRK-52E cells (*n* = 3, *p < 0.05, **p < 0.01) **B** Co-localization of CX3CR1, NLRP3, and ST2 in the mouse kidney. 400×; scale, 20 µm. **C** Molecular docking of HNK and NLRP3. **D** The binding sensorgram (for the interactions between HNK and NLRP3).

Evidence suggests that ST2 can localize to renal tubules and the interstitial space, and the IL-33/ST2 pathway exacerbates the renal structural and functional damage in CKD. To further explore the effect of pyroptosis in renal resident macrophages on renal tubular epithelial cells, we performed the co-culture experiments described in the “Materials and methods” section and observed that HNK could reduce the protein level of ST2 in rat renal tubular epithelial cells (Fig. 7A). Immunofluorescence analysis of mouse renal sections also showed that HNK significantly downregulated NLRP3 and ST2 in renal resident macrophages and renal tubules, respectively (Fig. 7B). The molecular docking result demonstrated that Tyr632 interacts with HNK via an arene-H bond, and Arg578 establishes a hydrogen bond with HNK (Fig. 7C). To confirm that HNK binds to NLRP3, surface plasmon resonance (SPR) experiments were conducted. The equilibrium dissociation constant (KD) of HNK and NLRP3 was estimated at 28.65 ± 1.7 µM, respectively, indicating that HNK binds to NLRP3 directly (Fig. 7D).

## Discussion

SLE is characterized by an exaggerated pro-inflammatory response with a loss of immune tolerance, and LN is the most common complication of the disease. In the past few decades, the pathogenesis of LN has been shown to involve genetics, interferon signaling, potentially specific antigens, immune-cell dysfunction, and complement dysregulation [18]. Currently, the available therapeutic agents against LN are mainly corticosteroids, antimalarials, and immunosuppressive agents. All of these agents have significant side effects and provide limited improvement of renal injury even though they do improve the prognosis of LN patients [19]. Therefore, there is an imperative need for new strategies to develop alternative therapeutics against LN. *Magnolia officinalis* has been used as a traditional Chinese medicine for thousands of years. In recent years, HNK, the active component of *M. officinalis*, has been reported to have anti-inflammatory and therapeutic effects in various models of kidney diseases. However, the underlying mechanisms of HNK improves LN remains elusive.

MRL/lpr mice suffer from severe lymphoproliferative and renal diseases, which are serologically and pathologically similar to the symptoms observed in human SLE, characterized by the production of autoantibodies and autoimmune glomerulonephritis [20]. Thus, these mice are used as a classical animal model for studying SLE. Indeed, we observed that urinary protein, Scr, and BUN levels are significantly increased in MRL/lpr mice. Additionally, renal pathological staining showed inflammatory-cell infiltration around the glomerulus, basement-membrane thickening, and collagen deposition, and TEM suggested podocyte foot. Notably, all these pathological changes were significantly improved upon HNK intervention.

To explore the mechanism behind the improvement of LN by HNK, we analyzed our RNA-seq data using IPA and found that the mechanism of HNK intervention may be closely related to the regulation of the NLRP3 inflammasome pathway, and IL33 was at the center of the differential gene interaction network. The nucleotide-binding oligomerization domain-like receptor (NLR) family of proteins is a class of pattern-recognition receptors widely localized to the cytoplasm, where they function as sentinels of intracellular danger signals to initiate immune responses [21]. Among these proteins, “NACHT, LRR, and PYD domains-containing protein 3” (NLRP3) recognizes various pathogen-associated molecular patterns (PAMPs) or danger-associated molecular patterns and is associated with various inflammatory diseases in humans [22]. Previous studies in humans and various animal models have shown that the NLRP3 inflammasome plays an important role in the progression of SLE and LN [23–26]. For example, activation of NLRP3 inflammasomes was observed in kidney biopsies from patients with type IV LN as well as in the SLE–prone MRL/lpr mice and NZM2328 mice [27, 28], presenting the NLRP3 inflammasome as a therapeutic target in LN and SLE.

In addition, we screened weighted gene co-expression network analysis (WGCNA) and LN disease target databases for the DEGs derived from the RNA-seq results, and the results suggested that the IL-33 upregulation in MRL/lpr mice was significantly suppressed by HNK treatment. It is currently believed that the progression of kidney disease is closely related to the persistent activity of IL-33/ST2. Upon an infection or tissue damage in the kidney, IL-33 acts as a nuclear sentinel to sense the injury and then warns the neighboring cells and tissues. It induces immune-cell maturation and cytokine release and is involved in the initiation, maintenance, and regression of inflammation, as well as in the physiology and pathology of many diseases [29]. As an important subtype of ST2, ST2L is highly expressed in the kidney, lungs, and stomach [30]. A large amount of evidence suggests that ST2L is a functional component of IL-33 signaling, promoting inflammatory responses by activating pro-inflammatory factors, such as NF-κB [31]. There is increasing evidence that the IL-33/ST2 signaling pathway is involved in SLE, LN, and chronic kidney disease, as well as in the inflammatory pathogenesis of various kidney injury–related diseases [32]. Additionally, a study has recently found that the level of soluble ST2 is upregulated in SLE patients, compared with the level in healthy individuals, thus offering ST2 as a potential marker of LN [33]. Overall, emerging data support that the IL-33/ST2 pathway contributes to renal inflammatory response and chronic kidney injury.

In the present study, the single-cell data showed that pyroptosis-related genes are significantly upregulated in LN patients. Importantly, the GEO data and clinical samples from LN patients showed aberrant upregulation of NLRP3, IL-33, and ST2 in the kidneys with LN. In our animal experiments, HNK treatment suppressed the abnormal renal activation of the NLRP3 inflammasome and IL-33/ST2 pathways in MRL/lpr mice. In addition, the GEO chip data also showed a positive correlation between NLRP3 and *IL33*. Accordingly, we speculate a regulatory relationship between NLRP3 and IL-33 in renal resident macrophages.

Renal macrophages are a heterogeneous population of immune cells with distinct and opposing roles during kidney injury and repair, and macrophage origin can partially explain the heterogeneity of the macrophages in the kidney. Widespread evidence suggests that in many healthy organs, including the kidney, tissue-resident macrophages are thought to originate mainly from embryonic progenitors, with a significant increase in the renal infiltration of circulating monocytes derived from bone marrow, upon renal damage [34]. There is increasing evidence that resident macrophages play an important antecedent function to promote tissue health and homeostasis in diseases, such as inflammatory joint disease [35], myocardial infarction [36], and colitis [37]. Therefore, we used kTMs as a model, and our results show that NLRP3 directly activates the expression of IL-33. In addition, our co-culture experiments further demonstrated that there is a crosstalk between renal resident macrophages and renal tubular epithelial cells in LN. Results from molecular docking and SPR analysis showed that HNK could directly target NLRP3 and thereby modulate the NLRP3/IL33/ST2 axis and inhibit the intercellular crosstalk to improve LN.

Overall, our existing data suggest that there is an abnormal crosstalk between resident renal macrophages and renal tubular epithelial cells during the pathogenesis of LN, and these cells undergo activation of the NLRP3 inflammasome and IL-33/ST2 pathways, respectively. In addition, NLRP3 may directly regulate IL-33 expression, and HNK presumably ameliorates LN by inhibiting the NLRP3/IL-33/ST2 pathway.

## Conclusion

**Fig. 8:**
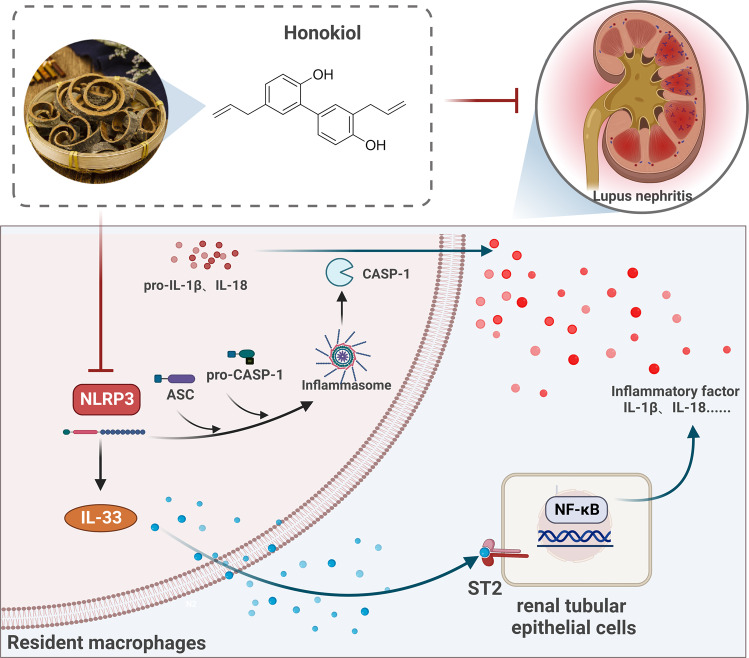
The mechanism underlying the therapeutic effect of Honokiol (HNK) against lupus nephritis. HNK decreased renal inflammation in the kidney by inhibiting the NLRP3/IL-33/ST2 pathway. Created using BioRender.com.

By using RNA-seq alongside IPA, this study identified core mRNAs and regulatory pathways that are likely involved in the anti-LN effect of HNK. Both the NLRP3 inflammasome and IL-33/ST2 pathways appear to aggravate the inflammatory responses in LN, and HNK significantly suppresses the activation of the NLRP3/IL-33/ST2 axis in LN patients (Fig. 8). Accordingly, our study provides novel insights into the mechanisms underlying the therapeutic effect of HNK on LN, thereby paving the way for the development of new therapeutic strategies against LN.

## Materials and methods

### Chemicals and reagents

The reagents used in this study are as follows: Drugs Honokiol (MCE,CAT#HY-N0003, purity is 99.90%), ATP (MCE,CAT#HY-B0345A/CS-2387), ASC oligomerization inhibitor MCC950 (MCE,CAT#HY-12815A), Caspase-1 inhibitor VX-765 (MCE,CAT#HY-13205), LPS (Sigma,CAT#L2880), collagenase IV (BioFroxx,CAT#2091MG100), DNase I (SAITONG,CAT#PS0825), single-cell suspensions kit (Solarbio,CAT#P5380), RPMI-1640 media (Gibco, REF:C11875500BT), FBS (Multicell, CAT NO:086-150), p3*flag-NLRP3 (PPL, CAT#PPL00151-2b), P3 Primary Cell 4D-Nucleofector X Kit L (Lonza,CAT#V4XP-3024), Bradford Assay kit(Beyotime,CAT#P0006), Blood Urea Nitrogen (BUN) Detection Kit (Nanjing Jiancheng Bioengineering Institute,CAT#C013-2-1), Creatinine Assay Kit (Nanjing Jiancheng Bioengineering Institute,CAT#C011-2-1), IL-33 (Affinity,CAT#DF8319),NLRP3 (Affinity, CAT#DF74), PrimeScript RT Reagent Kit (Takara, CAT#RR037A), HieffTM qPCR SYBR Green Master Mix (YEASEN, CAT#11201ES08), RIPA lysis buffer (Beyotime,CAT#P0013B), Nuclear protein Extraction Kit (Solarbio,CAT#R0050), BCA Protein Assay kit (Beyotime, CAT#P0012), PVDF membranes (Millipore,CAT#ISEQ00010), NLRP3 (CST,CAT#20836T), Cleaved caspase-1 (Affinity,CAT#AF4005), Cleaced IL-1β(CST,CAT#20839T), Caspase-1 (CST,CAT#20842T), ASC (CST,CAT#20838T), IL-33 (Affinity,CAT#DF8319), ST2 (proteintech,CAT#60112-1-Ig), NF-κB (Affinity,CAT#BF8005), Tublinβ (Affinity,CAT#AF7011), Lamin B (Affinity,CAT#BF8009), GAPDH (Abbkine,CAT#KTD101-CN), secondary rabbit-antibody (Immunoway, CAT#RS23920), secondary mouse-antibody (Abbkine,CAT#A23710), Mouse IL-33 ELISA Kit (MultiSciences,CAT#70-EK233/2-48), Mouse IL-1β ELISA Kit (FANKEWEI,CAT#F2923-B), Rat IL-1β ELISA Kit (FANKEWEI,CAT#F2923-B), Rat IL-18 ELISA Kit (FANKEWEI,CAT#F3070-B). Recombinanl NLR Family, Pyrin Domain Containing Protein 3(NLRP3) (Cloud-Clone, CAT#RPK115Hu01).

### Animals and experimental design

Specific-pathogen–free female MRL/lpr and MRL/MpJ mice (6–8 weeks old) were raised in the Animal Experiment Center of Zhejiang Chinese Medical University (Hangzhou, China). These mice were housed under standard environmental conditions (22 ± 2 °C, 40–60% relative humidity, and 12 h/12 h light/dark cycle) and provided with food and water ad libitum. The MRL/lpr mice were randomly divided into three groups—the model group (*n* = 6), the HNK group (*n* = 6), and the AAV-shRNA-NLRP3 group (*n* = 6). The MRL/MpJ mice were used as the Control group (*n* = 6). After adaptive feeding for a week, the HNK group was treated with HNK (40 mg/kg per day, i.g.), and the other groups received distilled water (0.1 ml/animal per day, i.g.). Afterward, urine samples and the kidneys were collected. The eyeballs were removed for blood collection. The AAV2/8 viruses used for the AAV-mediated shRNA expression in this study were diluted in phosphate-buffered saline (PBS) and injected via the tail vein. Through this strategy, 2×10^10^ vector genomes/g body weight can provide efficient renal transduction. The AAVs carrying the NLRP3-targeting shRNA were obtained from OBiO Technology (Shanghai, China). The shRNA sequences were 5′- GGCCTTACTTCAATCTGTT-3′.

### Grouping and transfection of renal resident macrophages

Clean SD rats were sacrificed via cervical dislocation under sterile conditions, and the kidneys were extracted. Afterward, the visceral fat and renal capsule were stripped off, and the kidneys were minced into small pieces (<1 mm^3^), which were subsequently digested in Hank’s buffered salt solution with 0.5 mg/mL collagenase IV and 0.1 mg/mL DNase I at 37 °C for 30 min with shaking. The digest was filtered through a 70 μm mesh filter to obtain single-cell suspensions of renal cells, which were subsequently washed with PBS. Renal resident macrophages were isolated from the single-cell suspensions by using a kit according to the manufacturer’s instructions. Subsequently, the cell concentration was adjusted to 10^6^/mL in RPMI-1640 media containing 10% fetal bovine serum, and then the cells were seeded into T25 cell-culture flasks. After 3 h of culture, the non-adherent cells were removed, and the renal resident macrophages (kTMs) were sorted out.

kTMs were cultured with or without lipopolysaccharide (LPS, 10 µg/mL) for 24 h + 5 mM ATP for 1 h. Then, the cells were divided into seven groups as follows: (A) the blank control group; (B) the flag-NLRP3 group; (C) the negative control group (NC, transfected with empty vector); (D) the stimulation group (treated with LPS + ATP); (E) the stimulation + JP group (treated with LPS + ATP and 10 µg/ml HNK); (F) the stimulation + si-NC group (treated with LPS + ATP and then transfected with a non-targeting control siRNA); (G) the stimulation + si-IL-33 group (treated with LPS + ATP and then transfected with an IL-33 siRNA). Transfection of the cells with an NLRP3-expression plasmid, IL-33 siRNA, or the corresponding controls was performed using Amaxa™ 4D-Nucleofector (Lonza) as follows: 5 × 10^5^ cells per reaction were resuspended in 100 µl of the nucleofection solution for primary cells of P3 Primary Cell 4D-Nucleofector X Kit L (Lonza). Immediately after the nucleofection, 500 µl of pre-warmed RPMI-1640 medium was added to the cells, which were then gently transferred to six-well plates containing 2 ml pre-warmed medium per well. After 48 h, the cells were lysed and their total-protein concentrations were measured for the subsequent western blot analyses. The details of the plasmid and siRNA are as follows: IL-33 siRNA, 5ʹ-GCUCUGGCCUUAUGAUAAATT-3ʹ and 5ʹ-UUUAUCAUAAGGCCAGAGCTT-3ʹ. The small-interfering RNA (siRNA) against the *IL-33* RNA was purchased from GenePharma (Shanghai, China), and p3*flag-NLRP3 was obtained from PPL (Public Protein/Plasmid Library, China)

### Co-culture experiments

Rat renal tubular epithelial cells (NRK-52E cells) were obtained from ATCC. They were divided into five groups as follows: (A) NRK-52E–alone group; (B) kTMs co-cultured with NRK-52E; (C) kTMs transfected with flag-NLRP3 and then co-cultured with NRK-52E cells; (D) kTMs transfected with si-IL-33 and then co-cultured with NRK-52E cells in the presence of JP-treated serum; (E) kTMs co-transfected with si-IL-33 and flag-NLRP3 and then co-cultured with NRK-52E cells. Co-culture was performed using transwells (0.4-μm pore size, Corning), and cells were collected after 48 h of co-culture.

### Assessment of renal function

Urinary protein, blood urea nitrogen (BUN), and serum creatinine (SCr) levels were measured to evaluate renal function. Bradford assay was used to determine urinary protein concentration. BUN Detection Kit and Creatinine Assay Kit were used for determining BUN and SCr levels. The spectrometric absorbance of each sample was measured using a microplate reader and used to calculate the concentration of each index according to the standard curve.

### Renal pathology

#### Histopathological analysis of the mouse kidney samples

Mouse kidney samples were dehydrated, paraffin-embedded, and sectioned (4–5 µm) according to the standard methods. Then hematoxylin-and-eosin (HE), periodic acid silver methenamine (PASM), and Masson staining were performed. Finally, the stained samples were analyzed under a light microscope.

#### TEM analysis of the mouse kidney samples

After isolating the kidney samples, they were immediately embedded in tissue blocks of ≤3 mm^3^. The tissue blocks were immediately fixed in the electron microscopy fixative for 2 h at room temperature and then transferred to 4 °C for subsequent experiments. Next, the tissues were fixed with 1% osmium tetroxide for 2 h, dehydrated through an ethanol gradient, washed with acetone, and then embedded within Epon 812. Finally, each tissue block was cut into 60 nm ultrathin sections, stained with uranyl acetate and lead citrate, and then observed via TEM.

#### Immunofluorescence analysis of the human and mouse kidney samples

Paraffin-embedded kidney sections (5 μm) were incubated with anti-IL-33, anti-NLRP3, anti-CX3CR1, and anti-ST2 antibodies. Finally, a fluorescence scan or fluorescence microscopic photo observation was performed.

#### Immunohistochemical analysis of the human kidney samples for the IgG level

Paraffin sections were de-waxed. antigen retrieval, blocking of endogenous peroxidase, serum blocking, incubation with IgG primary antibody, secondary antibody incubation, coloration, restaining of nuclear, dehydration, and sealing. Then, microscopy was performed, and images were collected and analyzed.

### RNA-seq and bioinformatics analysis

Total RNA was isolated using a TRIzol reagent. RNA concentration, purity, and integrity were assessed, and quality control was carried out. Then, the mRNA content was enriched and fragmented. cDNA was synthesized using random hexamer primers, followed by end-repair, tailing, adapter ligation, and polymerase chain reaction (PCR) enrichment. Finally, Illumina high-throughput sequencing was performed. The above steps were performed by Lianchuan Biotechnology Co.Ltd. (Hangzhou, China). Then, we screened the data for the differentially expressed genes (DEGs, *p* value <0.05 and |log_2_ (fold change) |≥2), which were subsequently uploaded to the ingenuity pathway analysis (IPA) software for core analysis.

### Gene expression analysis

To identify the major DEGs between the healthy and LN human kidney samples, we mined the GEO database (http://www.ncbi.nlm.nih.gov/geo/). The datasets we selected had quite a few samples and appropriate microarray annotation data, including those involving glomerulus and renal tubules. DEGs were defined using the limma package in the R statistical environment. A *p* value <0.01 and a threshold value ≥1.5 for fold change |FC| were applied.

### WGCNA

The weighted gene co-expression network was constructed using the R language of the WGCNA package. The pickSoftThreshold function was used to obtain the optimal value of the adjacent function weighting parameters, which is used as a soft threshold for subsequent network construction. A weighted adjacency matrix was then generated, and related gene modules were constructed based on the hierarchical clustering of the dissimilarity measure (1-TOM) of the topological overlap matrix (TOM). Finally, the expression profiles of each module were summarized using the module eigengene (ME), and the correlation between the ME and each clinical feature was assessed. Therefore, the modules highly correlated with the phenotypic features were chosen and the genes in these modules were selected for subsequent analyses.

### Collection of LN disease targets

To further clarify whether HNK regulates LN disease targets, we collected LN targets from the database. For example, by using “lupus nephritis” as the keyword, relevant targets were obtained from the following four databases: (1) DrugBank (http://www.drugbank.ca/,version: 4.3). (2) OMIM (http://www.omim.org/, last updated on 10 April 2016). (3) GenCLip (http://ci.smu.edu.cn/genclip3/analysis.php). (4) PharmGKB (https://www.pharmgkb.org/index.jsp, last updated on 7 April 2016). Finally, targets that are mentioned in at least two or more two of the databases were included and extracted.

### Single-cell data mining from LN patients

Publicly available single-cell sequencing data from the kidney biopsies of 24 LN patients and ten healthy (control) individuals (Arazi A, Rao DA, Berthier CC, Davidson A, Liu Y, Hoover PJ, et al. The immune cell landscape in kidneys of patients with lupus nephritis. Nat Immunol. 2019 Jul;20 [7]:902-914.https://www.immport.org/shared/study/SDY997) were downloaded. After combining the samples, the Seurat package was used to construct single-cell analysis objects. The parameter min. features were set to ensure that the expression levels of ≥200 genes were available per cell and that the expression level of each of these genes was available in ≥10 cells. Dimensionality reduction was performed using UMAP. In addition, we selected KEGG and other sources to identify a pyroptosis-related gene set and then conducted gene set enrichment analysis (GSEA) on this gene set.

### Reverse transcription–quantitative PCR (RT-qPCR)

TRIzol reagent was used to extract total RNA, and PrimeScript RT Reagent Kit was used for the RT reaction. Subsequently, the resulting cDNA was subjected to qPCR analysis by using HieffTM qPCR SYBR Green Master Mix and CFX96TM real-time PCR system (Bio-RAD, CA, USA). The primer sequences were as follows: IL-33, 5ʹ-TTGGATGAGATGTCTCGGCT-3ʹ and 5ʹ-GTTTCCAGAGGAATGACGCA-3ʹ; NLRP3, 5ʹ-GACCAGCCAGAGTGGAATGAC-3ʹ and 5ʹ-CTGCGTGTAGCGACTGTTGAG-3ʹ; and GAPDH, 5ʹ-GTGTTCCTACCCCCAATGTGT-3ʹ and 5ʹ-ATTGTCATACCAGGAAATGAGCTT-3ʹ. The relative expression levels of the target mRNAs were calculated using the 2^−∆∆CT^ method and then normalized.

### Western blotting

Kidney and cell samples were lysed using the RIPA lysis buffer, and the nuclear proteins were extracted using a nuclear protein extraction kit. The protein concentrations of the lysates were determined using a BCA Protein Assay Kit. Next, 20 µg total protein per sample was resolved using sodium dodecyl sulfate–polyacrylamide gel electrophoresis (SDS-PAGE) and then transferred onto 0.2 µm PVDF membranes by using a semidry Trans-Blot apparatus (BIO-RAD, Hercules, CA, USA). Subsequently, the membranes were blocked at room temperature (20–30 °C) for 1 h with 5% skim milk in Tris-buffered saline containing 0.1% Tween-20 (TBST). Afterward, the membranes were incubated overnight at 4 °C with the following primary antibodies: antibodies against NLRP3, cleaved caspase-1, cleaved IL-1, caspase-1, ASC, IL-33, ST2, NF-κB, tubulin β, lamin B, and GAPDH. Next, the membranes were washed three times with TBST and then incubated at room temperature for 2 h with a secondary anti-rabbit or anti-mouse antibody, as required. The blots were analyzed using an Odyssey fluorescence scanner (LI-COR, Biosciences, Lincoln, NE). The signals were captured by using the supplied Odyssey software v3.0, and the results were expressed as fold changes normalized to the expression levels of tubulin, GAPDH, or lamin B.

### ELISA

By following the manufacturer’s instructions, we prepared kidney lysates and serum samples from mice. Mouse IL-33 ELISA Kit was used to quantitate IL-33 in the kidney lysates and sera, Serum IL-1β levels were determined using Mouse IL-1β ELISA Kit. For the culture supernatants, Rat IL-1β ELISA Kit and Rat IL-18 ELISA Kit were used.

### Computer docking

The 3D protein model of NLRP3 was downloaded from the Protein Data Bank (PDB, PDB entry code: 7PZC). Hydrogen atoms were added to the protein by using the modeling suite molecular operating environment (MOE) before carrying out the docking studies. By minimizing the contacts for hydrogen, the structures were subjected to an Amber99 energy minimization protocol. The structure of HNK was minimized, the partial charges were calculated using the MMFF94s force field, and all the possible ionization states were generated at pH 7.0 by using the MOE suite. HNK was docked into the 3D model using the MOE software; the binding site of the inhibitor was modeled based on PDB 7PZC information [38]. The default Triangle Matcher was used as the placement method, followed by force-field refinement, and London dG scoring was used for the docking. The top-scoring conformation of the compound was kept for analysis. Ligand interactions were generated using Chimera [39].

### Surface plasmon resonance (SPR) assay

The binding assays based on the SPR technology were performed in a Biacore 8 K instrument (GE Healthcare) at 25 °C by using PBST (PBS, pH 7.4, containing 0.005% Tween-20) as the running buffer. The protein sample was dissolved in the coupling buffer (20 μg/mL in 10 mM sodium acetate [pH 5.0]) and then immobilized onto a CM5 chip that had been equilibrated with PBST overnight. HNK (60 μM) was serially diluted and then injected at a flow rate of 30 μL/min for 100 s (contact phase) followed by 80 s (dissociation phase). The binding data were collected using the Biacore 8 K evaluation software (GE Healthcare).

### Statistical analysis

Experimental data were expressed as mean ± SD. GraphPad Prism software (version 8.0.1 for Windows) was used for the statistical analyses. Statistical significance was determined using ANOVA. All the experiments were successfully repeated at least three times.

## Supplementary information

Reproducibility Checklist

Author Contribution Statement

Approval Letter of Ethics Review Committee of The Second Affiliated Hospital of Zhejiang Chinese Medical University

patient’s clinical information-healthy control

patient’s clinical information

Original Data File

CERTIFICATE OF ENGLISH EDITING

## Data Availability

The datasets used and/or analyzed during the current study are available from the corresponding author on reasonable request.
