# Supplementary material for: Honokiol suppresses the aberrant interactions between renal resident macrophages and tubular epithelial cells in lupus nephritis through the NLRP3/IL-33/ST2 axis
Source: Cell Death Dis. 2023 Mar 1;14(3):174. doi: 10.1038/s41419-023-05680-9 (PMC9977833; doi:10.1038/s41419-023-05680-9)
Supplement: Supplementary file 2 — Author Contribution Statement [file 41419_2023_5680_MOESM2_ESM.docx]

**Author Contribution Statement**

Q.M. and M.X. performed development of methodology and writing, review and revision of the paper; X.J, J.Q. and S.H. provided analysis and interpretation of data; H.Y., L.Y. and J.L. provided technical and material suppor; L.Z., Y.F. and P.Q performed study concept and design. All authors read and approved the final paper.
