# Supplementary material for: Honokiol suppresses the aberrant interactions between renal resident macrophages and tubular epithelial cells in lupus nephritis through the NLRP3/IL-33/ST2 axis
Source: Cell Death Dis. 2023 Mar 1;14(3):174. doi: 10.1038/s41419-023-05680-9 (PMC9977833; doi:10.1038/s41419-023-05680-9)
Supplement: Supplementary file 3 — Approval Letter of Ethics Review Committee of The Second Affiliated Hospital of Zhejiang Chinese Medical University [file 41419_2023_5680_MOESM3_ESM.pdf]

浙江中医药大学附属第二医院伦理委员会

涉及人的生物医学研究 伦理审查批件

Approval Letter of Ethics Review Committee of
The Second Affiliated Hospital of Zhejiang Chinese Medical University

批件号 Approval NO.：2021-KL-071-01

项目名称 Study Title	多组学联用探讨参与系统性红斑狼疮基本进程的生物标志物及解毒祛瘀滋阴方减毒增效的机制研究		
申办方 Sponsor			
受理号 Acceptance Number	2021-KL-071-01		
主要研究者 Principal Investigator	孙旗策	承担科室 Responsible department	风湿免疫科
审查类别 Category of Review	初始审查	审查方式 Type of Review	会议审查
审查日期 Date of Review	2021 年 12 月 10 日	审查地点 Location of Review	建科院 241 会议室
审查文件清单 Reviewed Items	1.初始审查申请报告<br>2.专家评审意见<br>3.研究方案（版本号：1.0；版本日期：2021-11-17）<br>4.免除、免签知情同意书申请表<br>5.研究者专业履历<br>6.研究利益冲突声明<br>7.主要研究者岗位职责<br>8.主要研究者责任声明		
审评意见 Evaluation	同意		
审查决定 Decision	委员会对该项目的审查决定为：☑同意（Approval）		
主任/副主任委员签字 Chair Signature	[signature]		
签发日期 Date of issue	2021 年 12 月 10 日		
伦理审查委员会 Stamp of IRB	伦理委员会盖章		
批件有效期 Period of Validity	自本伦理审查委员会初始审查批准之日起一年内，本临床研究应在本院启动。逾期未启动的，本批件自行废止。		
年度/定期跟踪审查 Continual Review	审查频率为该研究批准之日起每 12 月一次，首次，请于 2022 年 12 月 10 日前 1 个月递交研究进展报告。<br>伦理审查委员会有根据实际进展情况改变跟踪审查频率的权利。		

浙江中医药大学附属第二医院伦理委员会

声明 Statement	本伦理审查委员会的职责、人员组成、操作程序及记录遵循《涉及人的生物医学研究伦理审查办法》、《涉及人的健康相关研究国际伦理准则》、《赫尔辛基宣言》、GCP 和 ICH-GCP 等国际伦理指南和国内相关法律法规。

注意事项：

1.请遵循我国相关法律、法规和规章的伦理原则。

2.请遵循经本伦理审查委员会批准的临床研究方案、知情同意书、招募材料开展本研究，保护受试者的健康与权利。对研究方案、知情同意书和招募材料等的任何修改，均须得到伦理审查委员会审查同意后方可实施。

3.在本院发生的 SAE/SUSAR 以及研发期间安全性更新报告须按照 NMPA/GCP 最新要求及时递交我院伦理审查委员会，国内外其他中心发生的 SAE/SUSAR 需定期汇总、评估后递交伦理审查委员会。

4.根据报告情况，伦理审查委员会有权对其评估做出新的决定。

5.自今日起，无论研究开始与否，请在跟踪审查日到期前 1 个月提交研究进展报告。

6.申办方应当向组长单位伦理审查委员会提交中心研究进展报告汇总；当出现任何可能显著影响研究进行或增加受试者危险的情况时，请申请人及时向伦理审查委员会提交书面报告。

7.研究纳入了不符合纳入标准或符合排除标准的受试者，符合中止研究规定而未让受试者退出研究，给予错误治疗或剂量，给予方案禁止的合并用药等没有遵从方案开展研究的情况；或可能对受试者的权益或健康以及研究的科学性造成不良影响等违背 GCP 原则的情况，请申办方、监查员或研究者提交违背方案报告。

8.申请人暂停或提前终止临床研究，请及时提交暂停或终止研究报告。

9.完成临床研究，请申请人提交结题报告。

10.凡涉及中国人类遗传资源采集标本、收集数据等研究项目，必须获得中国人类遗传资源管理办公室批准后方可在本中心开展研究。

11.凡经我院伦理审查委员会批准的研究项目在实施前，申请人应按相关规定在国家卫健委、药审中心等的临床研究登记备案信息系统平台登记研究项目相关信息。

地址：杭州市拱墅区潮王路 318 号　邮编：310005　电话：0571-85267016　联系人：黄延彪

浙江省中医药大学附属第一医院伦理委员会
