# Supplementary material for: Honokiol suppresses the aberrant interactions between renal resident macrophages and tubular epithelial cells in lupus nephritis through the NLRP3/IL-33/ST2 axis
Source: Cell Death Dis. 2023 Mar 1;14(3):174. doi: 10.1038/s41419-023-05680-9 (PMC9977833; doi:10.1038/s41419-023-05680-9)
Supplement: Supplementary file 6 — Original Data File [file 41419_2023_5680_MOESM6_ESM.pptx]

## Slide 1
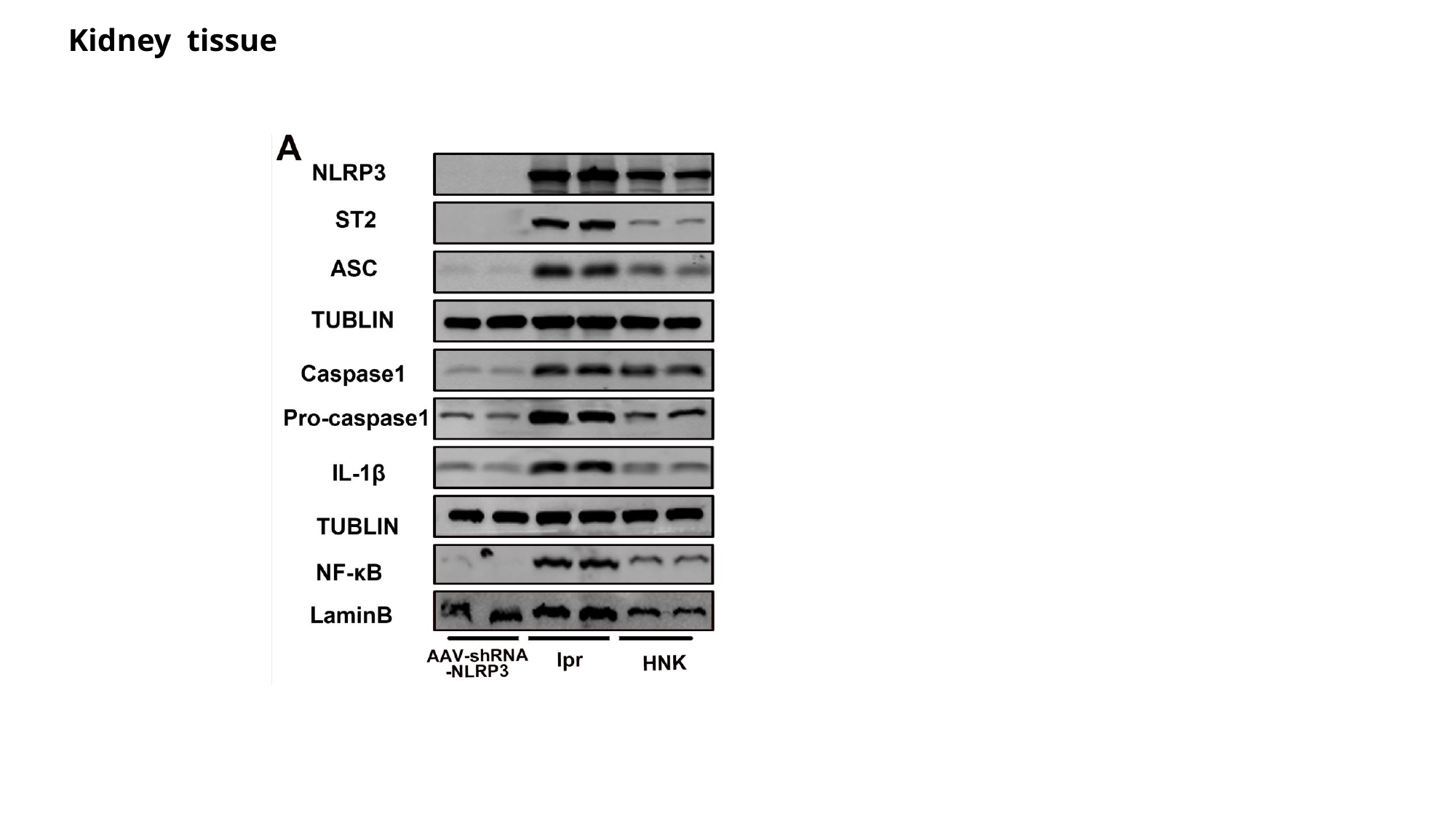

Kidney tissue

## Slide 2
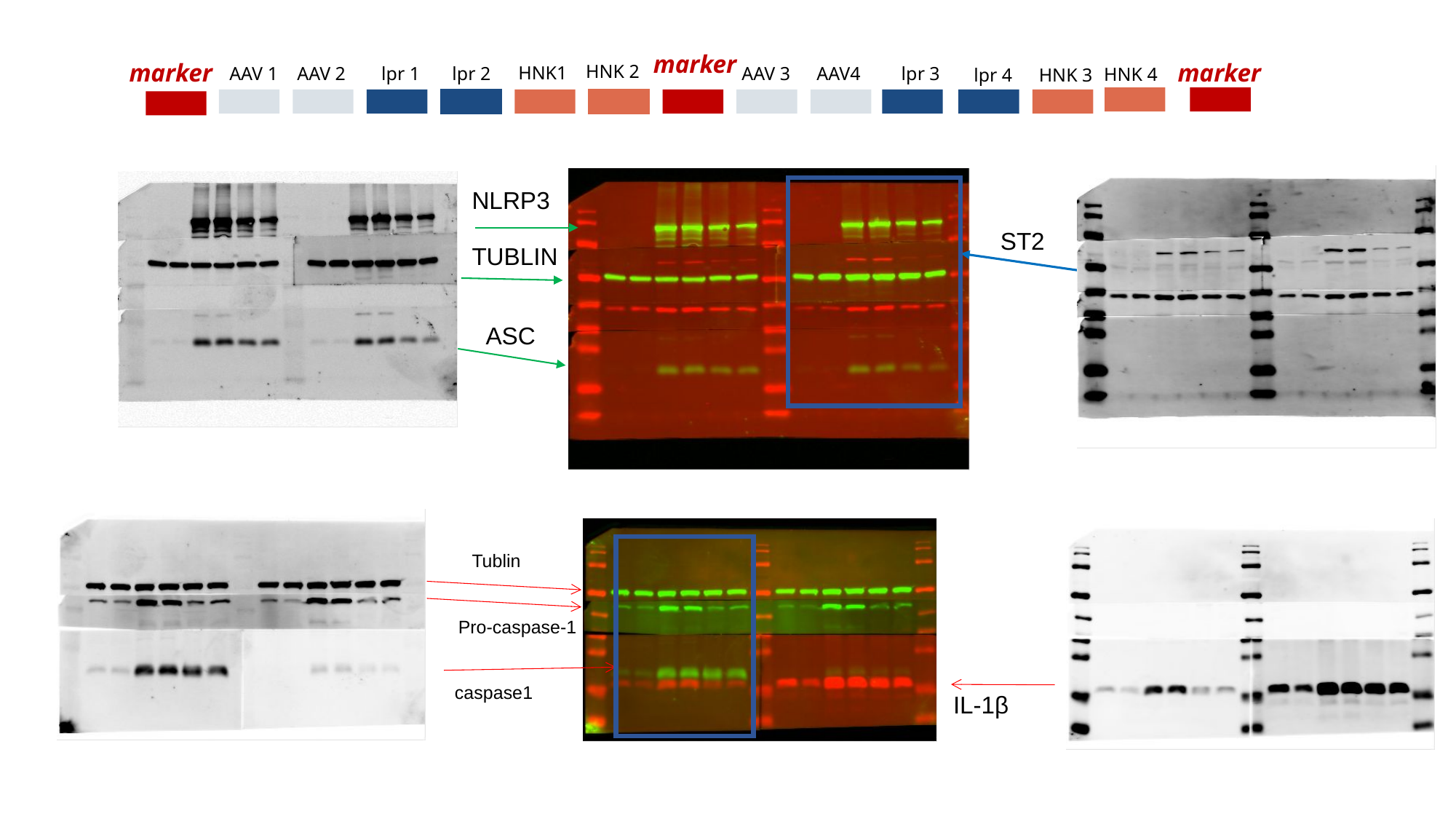

marker
 marker
 marker
AAV 1
AAV 2
lpr 1
lpr 2
AAV 3
AAV4
lpr 3
lpr 4
HNK 2
HNK1
HNK 4
HNK 3
NLRP3
ST2
TUBLIN
ASC
Tublin
Pro-caspase-1
caspase1
IL-1β

## Slide 3
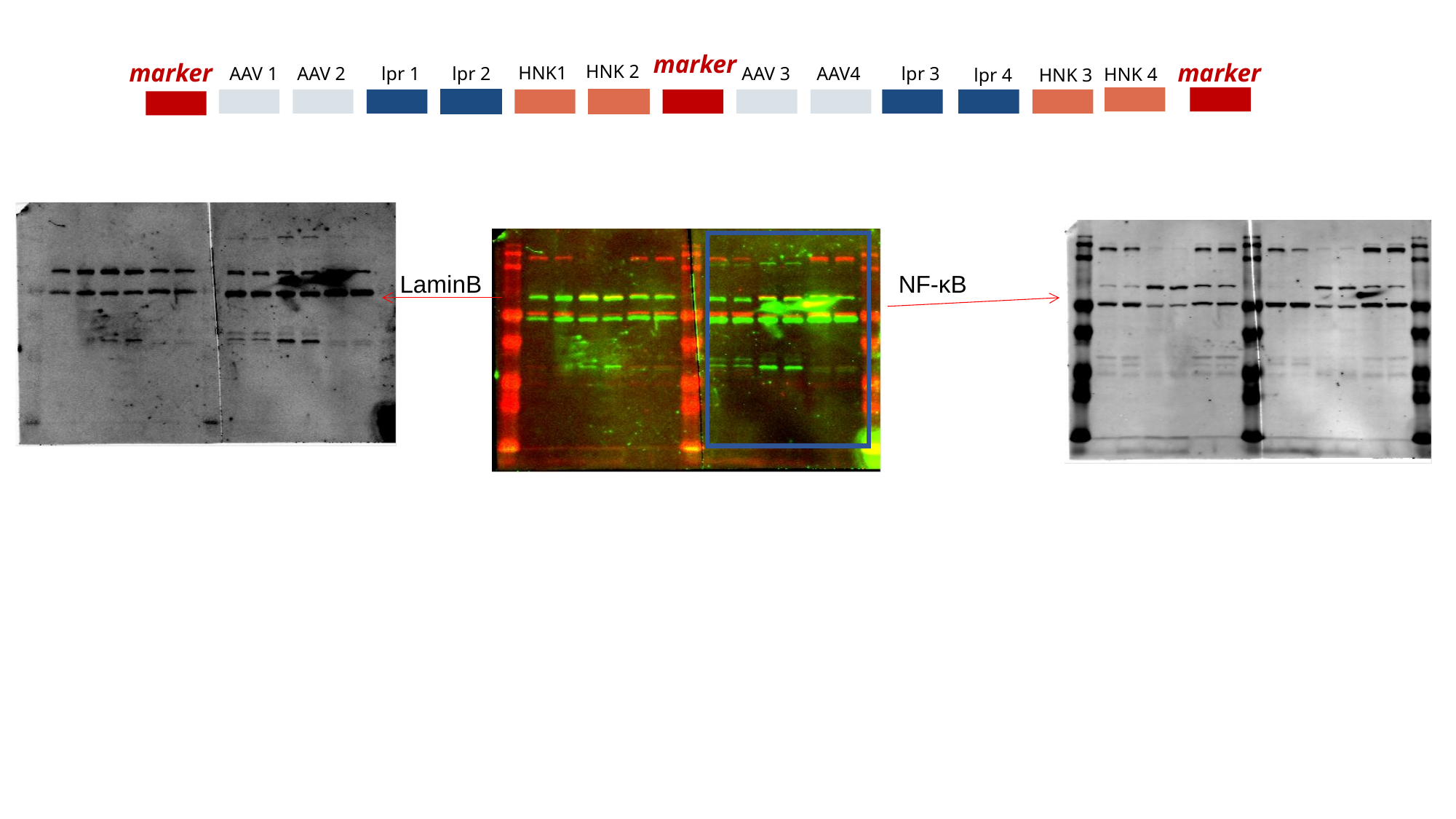

marker
 marker
 marker
AAV 1
AAV 2
lpr 1
lpr 2
AAV 3
AAV4
lpr 3
lpr 4
HNK 2
HNK1
HNK 4
HNK 3
LaminB
NF-κB

## Slide 4
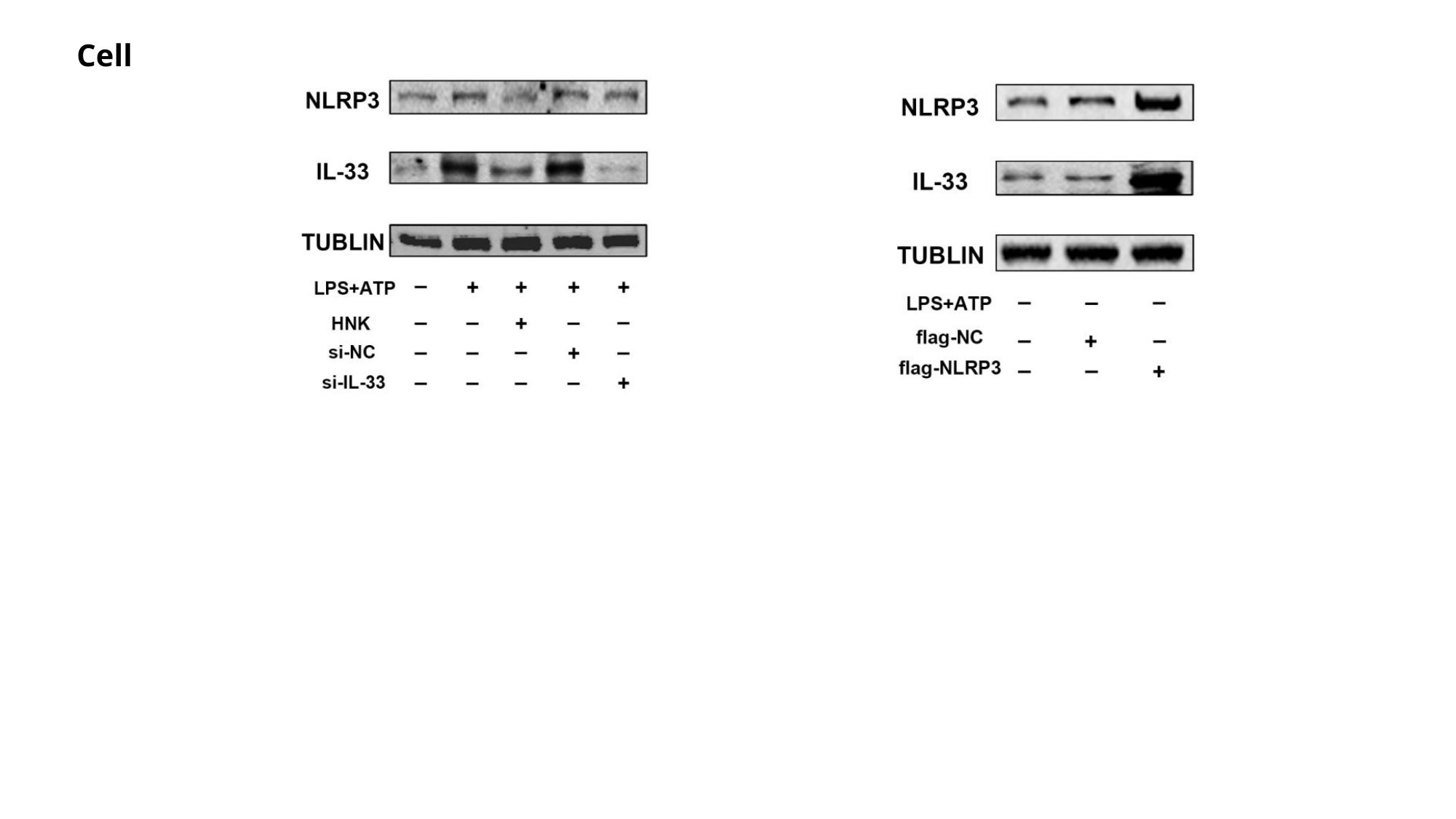

Cell

## Slide 5
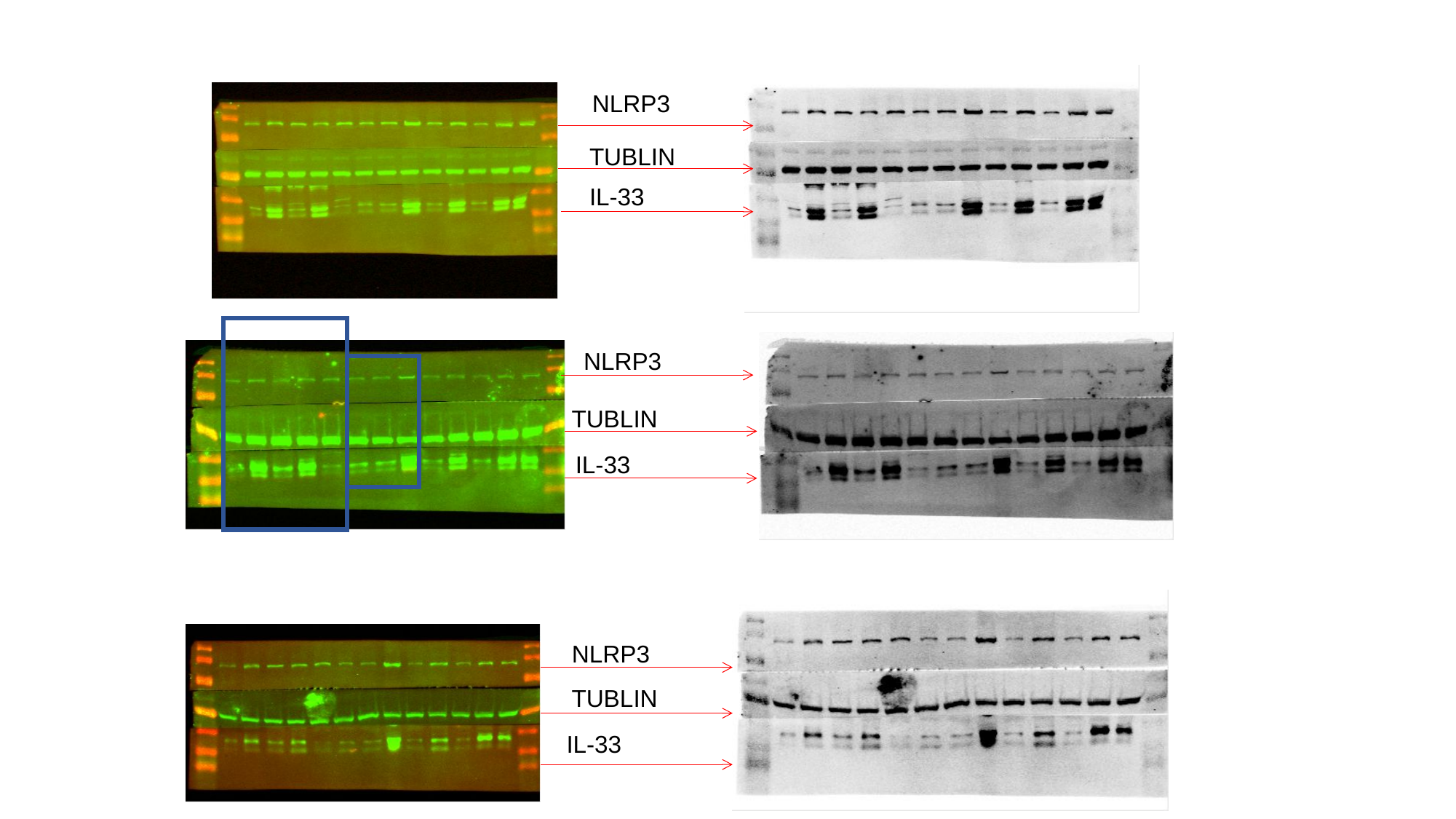

NLRP3
TUBLIN
IL-33
NLRP3
TUBLIN
IL-33
NLRP3
TUBLIN
IL-33

## Slide 6
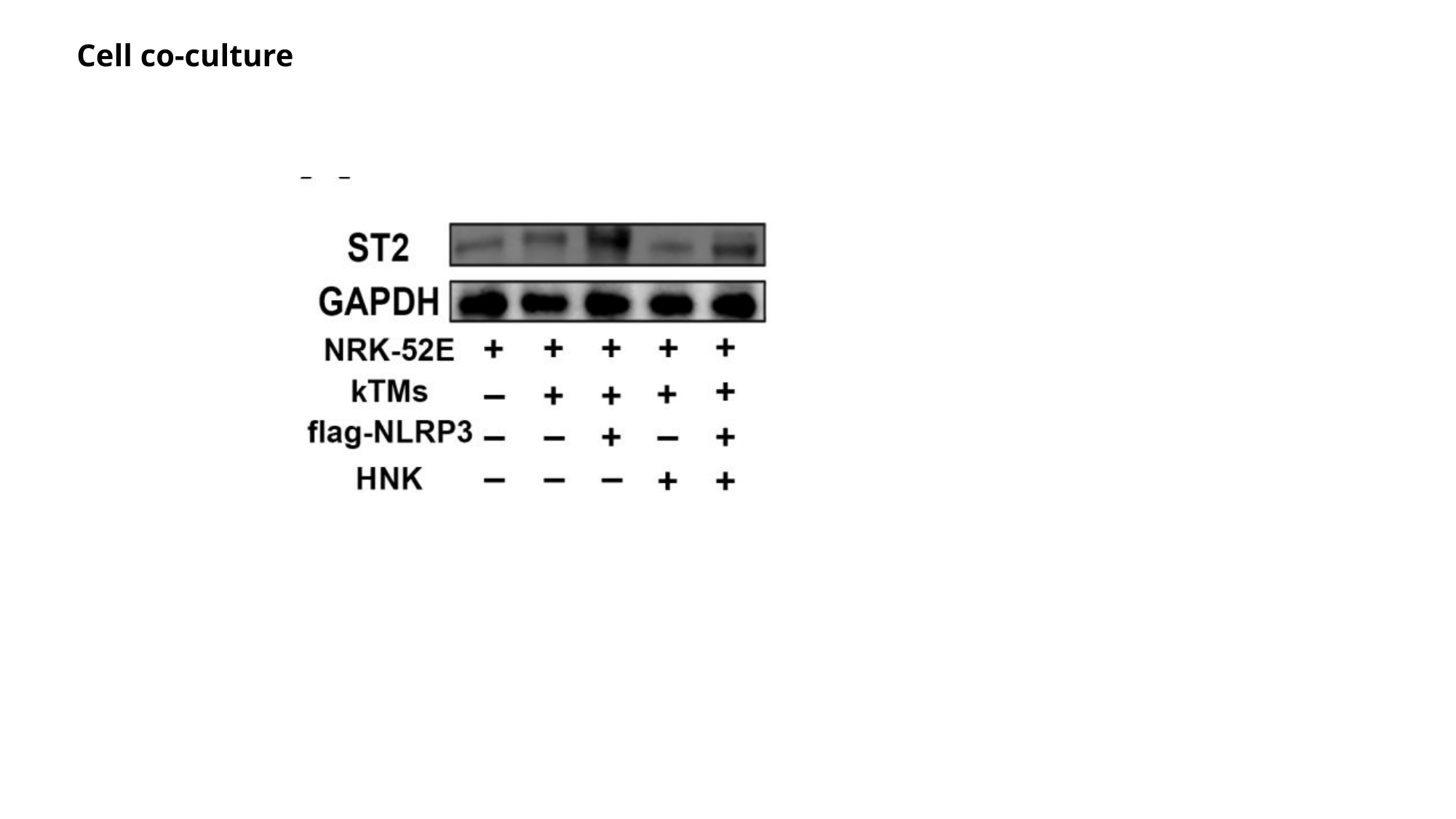

Cell co-culture

## Slide 7
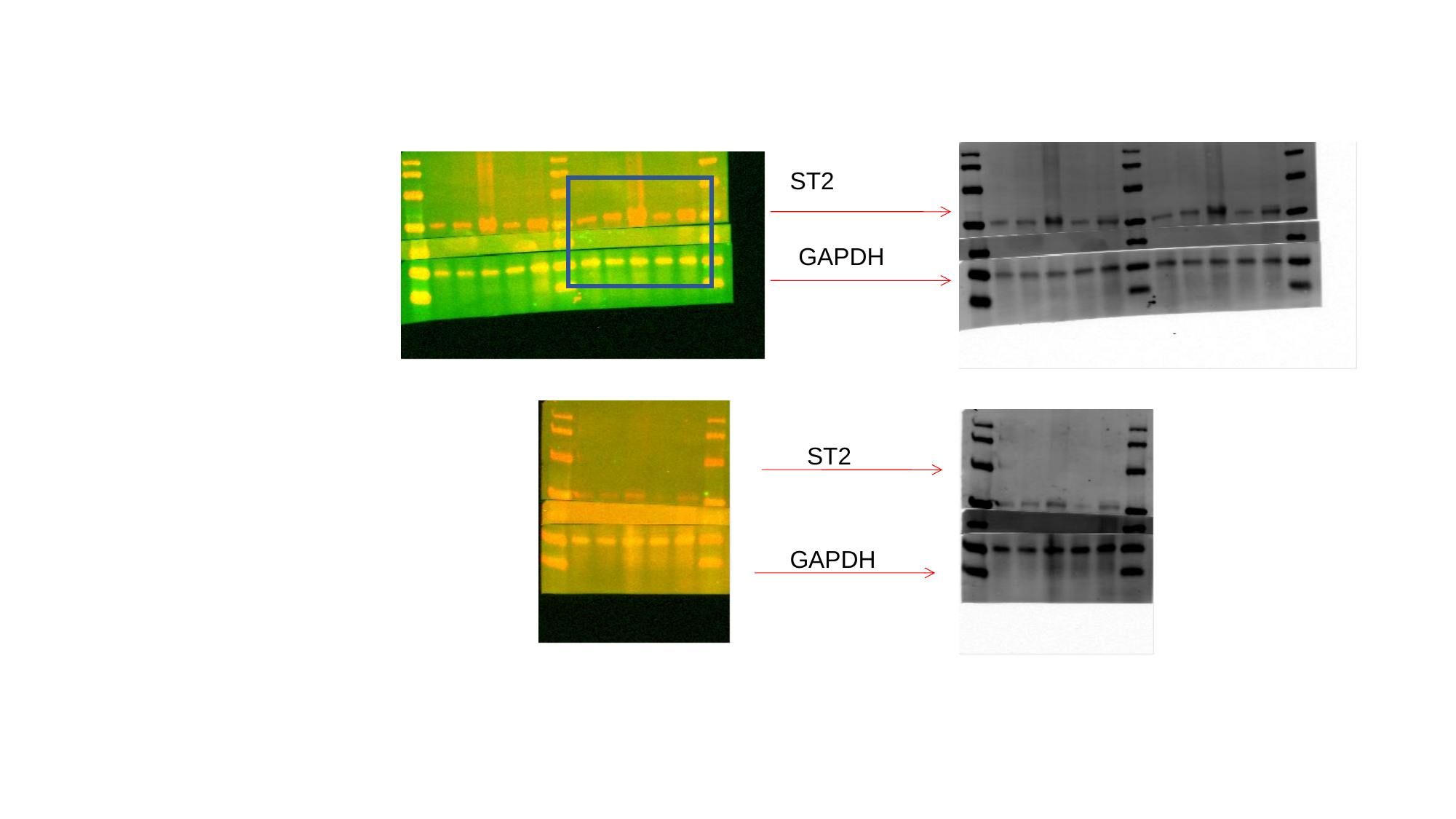

ST2
GAPDH
ST2
GAPDH
