# Supplementary material for: Honokiol suppresses the aberrant interactions between renal resident macrophages and tubular epithelial cells in lupus nephritis through the NLRP3/IL-33/ST2 axis
Source: Cell Death Dis. 2023 Mar 1;14(3):174. doi: 10.1038/s41419-023-05680-9 (PMC9977833; doi:10.1038/s41419-023-05680-9)
Supplement: Supplementary file 7 — CERTIFICATE OF ENGLISH EDITING [file 41419_2023_5680_MOESM7_ESM.pdf]

MogoEdit

CERTIFICATE OF ENGLISH EDITING

This is to certify that the manuscript entitled

Honokiol suppresses the aberrant interactions between renal resident macrophages and tubular epithelial cells in lupus nephritis through the NLRP3/IL-33/ST2 axis

commissioned to us has been carefully edited by a native English-speaking editor of MogoEdit, and the grammar, spelling, and punctuation have been verified and corrected where needed. Based on this review, we believe that the language in this paper meets academic journal requirements. Please contact us with any questions.

MOGOEDIT MOGO INTERNET TECHNOLOGY CO., LTD.

Gang Zhang

Dr. Gang Zhang
Founder & CEO of MogoEdit

Date of Issue
January 25, 2023

Disclaimer: The changes in the document may be accepted or rejected by the authors in their sole discretion after our editing. However, MogoEdit is not responsible for revisions made to the document after our edit on **January 25, 2023**.

MogoEdit is a professional English editing company who provides English language editing, translation, and publication support services to individuals and corporate customers worldwide. As a company invested by the affiliate fund of Chinese Academy of Science, MogoEdit is one of the leading language editing service providers in China, whose clients come from more than 1000 universities and research institutes.
MogoEdit Website: http://en.mogoedit.com/
500+ native English editors: http://en.mogoedit.com/editors

Mogo make the world flatter

Mogo Internet Technology Co., LTD.
No. 57, 3rd Keji Road, Xi'an 710075, PR China +86 02988317483 support@mogoedit.com
